# Practical quantitative measurement of graticule misalignment relative to collimator axis of rotation

**DOI:** 10.1120/jacmp.v11i4.3318

**Published:** 2010-08-15

**Authors:** Tarek Halabi, Bruce Faddegon

**Affiliations:** ^1^ Department of Radiation Oncology University of California San Francisco 1600 Divisadero St. San Francisco CA 94143‐1708 USA

**Keywords:** Graticule QA and replacement procedure

## Abstract

We design a practical procedure for measuring translational and rotational misalignment of graticule with collimator axis of rotation and collimator jaws, respectively. The procedure's quantitative results are accurate to less than 0.2 mm (at isocenter) and do not assume alignment of radiation focal spot with collimator axis of rotation. When provided with these quantitative results, the manufacturer can custom‐adjust graticules to the purchaser's collimator head.

PACS number: 87.56.Fc

## I. INTRODUCTION

Portal images are compared with simulation images to verify fidelity of beam‐patient geometry from simulation to treatment.^(^ ^1^ ^)^ Often the difficulty in this comparison is in finding a common reference.^(^ ^2^ ^)^ Graticules, both in simulators and treatment linacs, appear as “built‐in rulers” in the images and are regarded as the common frame of reference for the comparison. In addition to beam‐patient geometry discrepancy, graticules help reveal block discrepancy between simulation and treatment and, more importantly, allow one to distinguish between the two types of errors.^(^ ^3^ ^)^

A mechanical graticule^(^ ^4^ ^)^ typically consists of metal wires embedded in an acrylic plate. When placed in the collimator tray at a nominal collimator angle, the wires point radially from the source – one set lying in the beam's cross‐plane, and the other in the beam's in‐plane.

Improvements in accuracy of gantry rotation and other mechanical components of our linacs warranted a reduction to 1 mm of our threshold for graticule misalignment. If this threshold is exceeded, we order a new graticule. This reduction in threshold increased requirements on the QA procedure in two respects. First, graticule alignment is now expected to more quickly drift out of threshold and this warrants a practical procedure, given the required frequency of the test. Second, it is now desirable to custom‐adjust newly ordered replacement graticules to individual collimator heads. To this end, the procedure must provide accurate quantitative results. We may then return the replacement graticule to the manufacturer (Radiation Products Design) along with the measurement's results and request a custom adjustment. In the case of manually adjustable graticules, our procedure can be used to confirm alignment after manual adjustment.

Relative to which axis, however, should the graticule misalignment be measured? Since beam‐patient geometry fidelity is a major goal, it is intuitive to measure misalignment relative to the radiation central axis. In fact, Du et al.^(^ ^5^ ^)^ make this choice in their proposed procedure. From the point of view of a comprehensive quality assurance program, we prefer to measure misalignment relative to the collimator axis of rotation (CAR). CAR is well defined for any single gantry angle. Radiation central axis, however, may vary under collimator rotation for a fixed gantry angle if focal spot is not aligned with CAR. By choosing CAR instead of radiation central axis as the reference for our alignment test, we eliminate dependence of our results on focal spot alignment with CAR.

## II. METHODS

**Figure 1:**
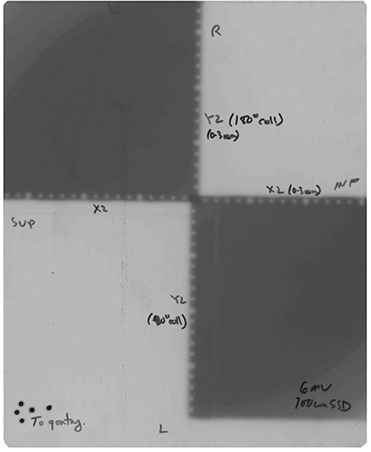
Double exposure film of graticule.

The general method of observing graticule misalignment with collimator axis of rotation is to take two exposures of the graticule with 180° collimator rotation between exposures. Regardless of focal spot alignment with CAR, the CAR is aligned with graticule center if and only if graticule dots of first exposure perfectly align with those of the second exposure. Generally, however, such a double exposed image simply consists of blurred graticule dots from which accurate quantitative measurement of misalignment may not be readily obtained. Therefore, in our procedure, each of the two exposures exposes a different quadrant of the film (same quadrant of graticule). On a Siemens Oncor linac: i) the X1 and Y1 jaws are fully opened, ii) X2 and Y2 jaws are opened to 0.3 cm, iii) the film is exposed, iv) collimator is rotated 180° and the film is exposed again. Ultimately, only the central dot is double exposed, and blurring is absent from the rest of the film (see Fig. 1). We use X‐OMAT V Film (33 cm×41 cm)) with 1 cm buildup and about 40 MU per exposure.

**Figure 2:**
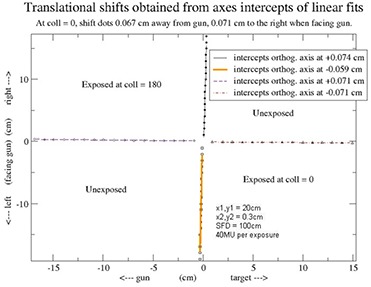
Shifts obtained from line fits to digitized graticule dots of film. Shifts apply at 0° collimator angle.

The exposures will appear shifted from each other, along the y‐axis, by twice graticule's translational misalignment along y‐axis. Similarly for the x‐axis. Henceforth, when we refer to positive and negative orientations along the x‐ and y‐axes, it is in reference to the first exposure's collimator setting. If dots of second exposure are shifted in the positive Y (X) direction relative to dots of first exposure, then the manufacturer must shift graticule grid in the positive Y (X) direction at first exposure's collimator setting. Shifts shown in Fig. 2 were computed this way. Furthermore, any rotation of the graticule relative to jaws is directly observed on film.

To obtain these shifts, the film is first scanned through a VIDAR scanner (VIDAR Systems Corporation, Herndon, VA). The resultant image file is processed using the software, ImageJ (National Institute of Health, Bethesda, MD). In this software, the dots are digitized and four straight lines are fit through each linear set of dots from each exposure (see Figure 2). The axes intercepts of these line fits are then used to obtain the shifts. If edges of secondary collimators are also digitized, graticule's rotational misalignment relative to these edges can also be quantified. However, we have not so far observed any significant rotational misalignment relative to the edges, and the step of digitizing collimator edges is left as optional. Finally, if rotational misalignment is measured, we recommend that the procedure follow mechanical checks of collimator rotation accuracy with a level. Our quality assurance of graticules follows a series of mechanical checks that include collimator rotation accuracy.

## III. RESULTS

Figures 1 and 2 were obtained from an application of the above procedure to a newly ordered replacement graticule. The difference between the two x‐axis intercepts, 0.074 cm ‐ (‐0.059 cm), divided by 2, provides the required 0.067 cm (at isocenter) shift away from gun when at 0° collimator angle. Similarly, the difference between the two y‐axis intercepts, 0.071 cm ‐ (‐0.071 cm), divided by 2, gives the required 0.071 cm (at isocenter) shift towards the right when facing gun.

The procedure's precision is limited by spatial resolution of the scanner and, to a lesser extent, the user's ability to accurately place mouse crosshairs at the center of graticule dots during digitization. In our application, scanner settings produced a half pixel width of 0.018 cm, and scanner resolution was the main source of error. In the worst case scenario, this error is oriented in the same direction for every dot in a linear set and would, therefore, be regarded as the systematic error in an intercept measurement. When the intercepts of the two exposures are summed, the systematic errors may add up or cancel. Again, we conservatively consider the worst case scenario and assume they add up to 0.036 cm. However, the shift is obtained by dividing this sum by 2, and our conservative estimate of systematic error in shift values becomes ≤ 0.018 cm at isocenter.

To gain further confidence in our assumption that, at this resolution, user error does not significantly add to the overall uncertainty, each of us independently carried out the digitization using a different scanner (but same film). Our results differed by 0.017 cm for one axis, and 0.009 cm for the other, within 1 pixel (0.018 cm) experimental uncertainty.

## IV. DISCUSSION

The X‐OMAT V Film, 33 × 41 cm, does not capture all graticule dots of one axis. SFD may be decreased to resolve this problem but this would come at too high a price of accuracy, which decreases with decreased magnification. A potential improvement would be to obtain film that is of similar quality but larger size. One can then even consider increasing SFD beyond isocenter to improve accuracy of shifts projected to tray position. It is possible that the 0.3 cm proximity of jaw shadow to that of graticule dots introduces systematic error in film development, and/or scanning/digitization. We may therefore benefit from opening jaws to 0.5 cm (instead of 0.3 cm noted above). Note also that we expose only one quadrant of graticule dots twice and that we may benefit from taking another double exposure film of the opposing quadrant. The most worthwhile measure to reduce uncertainty, however, would be to simply produce higher resolution scans.

## V. CONCLUSIONS

The procedure we have described is capable of validating graticule alignment with the collimator rotation axis and secondary collimators with an accuracy of better than 0.2 mm. It is useful for specifying the location of graticule dots when requesting custom adjustment from the manufacturer and for routine graticule QA. For the latter, software analysis is optional, and the user may confirm alignment by visual inspection of the films.
